# CHRONOFALLS: A multicentre nurse-led intervention in the chronoprevention of in-hospital falls in adults

**DOI:** 10.1186/s12912-023-01322-9

**Published:** 2023-05-05

**Authors:** Pablo Jesús López-Soto, Francisco José Rodríguez-Cortés, Rosa María Miñarro-Del Moral, María José Medina-Valverde, Rocío Segura-Ruiz, Pedro Hidalgo-Lopezosa, Roberto Manfredini, María Aurora Rodríguez-Borrego, CHRONOFALLS project collaborators, María Ángeles Ramírez-Pérez, Maria de la O Granados-Roldán, Francisco Javier Márquez-Cuenca, María Dolores Garrido-Ramiro, Celia Vicente-Fenoy, Juan de la Cruz López-Carrasco

**Affiliations:** 1 Department of Nursing, Instituto Maimónides de Investigación Biomédica de Córdoba (IMIBIC), Avda. Menéndez Pidal S/N, 14004 Córdoba, Spain; 2 Nursing Area. Faculty of Medicine and Nursing, Universidad de Córdoba, Avda. Menéndez Pidal S/N, 14071 Córdoba, Spain; 3 Department of Nursing, Hospital Universitario Reina Sofía de Córdoba, Avda. Menéndez Pidal S/N, 14004 Córdoba, Spain; 4 Faculty of Medicine, Pharmacy and Prevention, University of Ferrara, Via L. Ariosto 35, 44121 Ferrara, Italy; 5 Center for Studies On Gender Medicine, University of Ferrara, Via Fossato Di Mortara 64/B, 44121 Ferrara, Italy

**Keywords:** Accidental falls, Older people, Nursing care, Nursing, Temporal patterns, Prevention, Circadian rhythms

## Abstract

**Background:**

Falls are among the most common and serious adverse events for hospitalised patients. In-hospital falls pose a major medical and economic challenge for public health worldwide. Nevertheless, the issue is often addressed without regard to certain relevant variables such as the time of the fall. The aim of this study was to determine the effect of the implementation of a nurse-led intervention based on the temporal patterns of falls and their aetiology on the occurrence of falls.

**Methods:**

A mixed-method research design was carried out in three phases: a) a longitudinal prospective study (audits, chronobiological analyses and implementation of a multicentre nurse-led intervention based on temporal patterns of falls); b) a retrospective study of fall records; and c) a qualitative study based on focus groups. The protocol was published in 2021.

**Results:**

A difference was observed in the number of fall records before and after the chronopreventive intervention (retrospective: 64.4% vs. 35.6%; *p* < 0,001). According to the interrupted series analysis, considering the influence of the COVID-19 pandemic, a reduction in falls of 2.96% (95% CI 1.70%-4.17%) was observed. The concepts of falls, the COVID-19 pandemic and the causes of non-registration have emerged as categories for qualitative analysis.

**Conclusions:**

A multicentric nurse-led program based on tailored organisational, educational and behavioural chronopreventive measures seems to lead to a reduction in the number of in-hospital falls. The findings of the present study, highlighting the implementation of chronopreventive measures, can serve as a basis for future health policies.

**Trial registration:**

The project was registered on the Clinical Trials Registry NCT04367298 (29/04/2020).

**Supplementary Information:**

The online version contains supplementary material available at 10.1186/s12912-023-01322-9.

## Introduction

In recent decades, there has been an increase in life expectancy and a decrease in the birth rate, leading to a considerable ageing of the population [1]. This social circumstance has resulted in the need to preserve the autonomy and independence of this population group. Several studies [2–4] show that among the main causes of loss of autonomy and independence are falls, which constitute not only a social problem but also an economic problem [5, 6], due to the increased cost of health care and subsequent care arrangements required at home.

Numerous studies [7–9] have shown that the risk of falls increases with advancing age [1], i.e., it is proportional to the ageing of the population. Other studies [7, 10] indicate that 30% of institutionalized patients suffer a fall each year, and that this percentage rises to 40% in institutionalized elderly persons. As regards in-hospital falls, the incidence varies between 2–17 falls per 1000 occupied beds per day [9–11].

Hospital falls pose a risk to the patient's integrity [4], as they aggravate the different pathologies due to further damage caused to the body by impact. Moreover, as noted above, they diminish the patient's quality of life through loss of autonomy and independence. On many occasions, they also lead to an increase in the length of hospital stay and, consequently, an increase in healthcare costs [12–15].

In recent years, attempts have been made to identify the different risk factors involved in falls in order to preserve patient safety [16–18]. These factors are classified into two main groups [19, 20]: intrinsic factors (intrinsic to the person susceptible to falling), such as biological variables (age, sex, medical pathologies, adverse effects of medication, among others) or behavioural variables (lifestyle habits); and extrinsic factors (those that do not depend on the person themselves), such as socio-economic variables (quality of housing, salary, etc.) and environmental variables (pollution, lighting, etc.).

Among all the potential risk factors for fall causation, the seasonality of the falls has rarely been considered in incidence studies. In this regard, it is not only important to know the time of day at which the fall occurs, but also the work shift during which it occurs, and whether it occurs at the weekend or at a specific time of year.

Some studies [20–22] have shown that the time of day, the day of the week and the month of the year have a direct impact on the causality of the fall. However, despite demonstrating the importance of the temporal pattern in the cause, manner and place of the fall, only 50% of studies provided information about the time of the falls [23]. From a chronobiological point of view, preventive measures are needed, and the scientific area that deals with this is chronoprevention, which aims to reduce the number of falls by addressing the relative risk factors.

In recent years, a number of strategies have been implemented [16, 24] aimed at the prevention of in-hospital falls based on the timing of these falls. It was found that there is a morning peak in in-hospital falls around the time when patients are usually bathed or washed. Other studies [20, 25–27] reveal how the type of medication also influences the timing of falls.

Based on the above, time series analytical methods—used in the field of chronobiology to objectively detect and characterize biological rhythms—allow for a more complete understanding of the factors involved in the risk of falls, and can potentially be used in the future to develop effective preventive measures.

Consequently, the main objective of this study was to determine the effect of a tailored nursing intervention program on the temporal patterns of incidence and relative risk factors for falls and related injuries.

## Material and methods

### Design

We used a mixed design, with three approaches: (i) a prospective longitudinal design, with two follow-up periods of 18 months each; audits were conducted in both periods, also in the first period (January 2018—June 2019), the healthcare professionals attended seminars aimed at improving the recording methods of hospital falls, in order to identify both intrinsic and extrinsic factors specific to falls. In the second period (July 2019—December 2020), a multidimensional prevention program was implemented based on temporal patterns of falls, which focused on organizational, educational and behavioural elements for hospitalized persons and healthcare professionals; ii) a retrospective study of the falls recorded on the institutional databases of the hospitals studied during the assigned period (January 2018—December 2020); and iii) a descriptive exploratory design with a qualitative approach using focus groups, made up of healthcare professionals (nurses and nursing assistants) working in the hospitals studied. The study was registered on the Clinicaltrials.gov platform (NCT04367298) and a favourable report was from the relevant ethics committee (Act nº 270; ref. 3677).

The use of three different approaches is justified by the complex nature of in-hospital fall occurrences and its record. The quantitative part aims to describe all circumstances of the fall (what, who, how, where, why, and especially when) and the record by healthcare professionals (factors that may determine the record). Regarding the record by healthcare professionals, it was considered to understand the perceptions that could justify the record or not of the fall, and, therefore, explain the possible gap between the falls recorded retrospectively and prospectively.

Initially, the idea was to find out the impact of an intervention through a pre-post intervention experimental design. Following inconsistencies in the data gathered from study participants using a pre-post intervention approach, a mixed-model approach was implemented sequentially. A retrospective study was considered and, as the inconsistencies persisted, a qualitative approach using focus groups in each center were conducted.

### Participants and setting

The longitudinal study included information on hospitalized older people aged 18 years or above who had one or more falls during their stay in a tertiary-level referral hospital (university hospital) in southern Spain, and in three secondary-level hospitals (provincial hospital) in the same province, between January 2018 and December 2020. According to data from the Spanish Institute of Statistics, the population in 2018 covered by these hospitals was 786,524 people.

In the prospective study, information on the reference nurses attending to the person suffering from a fall was also considered in the study. The dependent variable was considered to be the presence of an in-hospital fall, defined as "the consequence of an unintentional and unexpected movement towards the ground from a higher position" [28].

In the retrospective phase, we took into account the institutional records of falls (from the four hospitals involved, during the same period) that occurred in hospitalized patients over 18 years of age.

Regarding the qualitative phase, the health professionals interviewed had worked in the four hospitals during the time of the longitudinal study. Participants, randomized selected (may or not be participants who registered falls in the prospective study), could be health professionals working in either: (i) clinical units where hospital falls were recorded during the longitudinal study; or (ii) where there was a significant risk of in-hospital falls (due to pathologies, dependence, motor or mobility disorder, etc.). The participation of hospital managers (supervisors or heads) was not considered.

### Instruments, data collection and procedure

For the longitudinal study (patients and nursing professionals), several instruments were used to collect data. Among these, the variables related to falls were collected by filling in a specific falls document, as described in the protocol [29]. Specifically, the Pittsburgh Sleep Quality questionnaire was used for sleep patterns; the Horne-Östberg Morningness-Eveningness questionnaire for chronotype; the SF-36v2 and GHQ28 questionnaires for health-related quality of life; and the Epworth Sleepiness Scale for variables such as sleepiness, fatigue and speed of patient response to the event.

Several instruments were used for retrospective data collection, in particular, the Minimum Basic Data Set and the patient safety register (adverse events register), and additional information was provided, after dissociation and anonymization, by the clinical and nursing care evolution and falls registers.

Prior to the longitudinal phase, several training sessions were held to explain to the healthcare professionals how the data collection was to be carried out and a specific shared folder was set up on the hospitals’ computer systems. During the study period, when a fall occurred, the circumstances of the fall had to be recorded, with the patient's prior consent, in order to gain access to the study variables. Following the analysis of the falls, preventive measures were implemented, focusing on the organization, education and behaviour of healthcare professionals. These measures were implemented in June-July 2019 in the four hospitals. Descriptive and time-series analysis of prospective and retrospective fall registration data and other characteristics associated with registration, together with the perceptions and impressions of the safety officers of the participating study sites (data obtained in the first phase seminars and audits), have determined the nature of the interventions. The countermeasures proposed to the centres are mentioned in Supplementary Material 1. Each centre implemented the measures considered appropriate. Nursing-led measures in which the time/timing of the implementation of each measure (*chronoprevention*) was decisive.

Regarding the focus groups, an open-ended script focused on fall-related factors was used (characteristics of falls – location, modality, cause, consequences and time of fall; professional evaluation; witnesses; treatment; recording and documentation; intervention; prevention and countermeasures; identification of risk; and difficulties). Five focus groups were held in the hospitals involved in the study following the longitudinal study (May–June 2021), all of which included at least five healthcare professionals. The focus groups were audio-recorded and lasted for 30 to 45 min. Two of the authors of this manuscript coordinated the focus groups, one as a moderator who guided the discussion, and the other to observe the body language of the participants and note any behaviour/attitudes that could not be captured on the recording. The healthcare professionals were informed and after giving their consent, only the sex and profession of each participant was registered.

### Data analysis

In the longitudinal and retrospective phases, descriptive statistics using SPSS were employed. A simple, multicomponent Cosinor analysis was performed to identify temporal patterns [30–32]. The CosinorPy-master package was used, using the Visual Studio Code software that employs the Python programming language (version 3.9.12). The residual sum of square was used to determine the best-fitting model and components. In addition, an interrupted time series analysis was carried out to determine the impact of the intervention, using the CausalImpact package in the RStudio software, which employs the R programming language (version 4.0.3). We also conducted an augmented Dickey-Fuller test, considering as a covariate the atmospheric temperature of a weather station close to the reference hospital. Specific analyses were also performed according to visualization, location, cause, position, and type of injury.

As the focus groups were audio-recorded, the audio was transcribed by two of the authors of this manuscript. After discussing the significance and interpretation of the transcriptions, a qualitative analysis was carried out into the conversation and discourse [33]. Bardin’s thematic content [34] was used to examine the “nuclei of meaning”, thus producing a message which might be significant for the analytical object studied. Three phases were performed: i) pre-analysis, to achieve a general impression of content; ii) exploration of the material, to encode the information in “registration units” which allow the accumulation of information in semantic categories; and iii) presentation of the results in a summary table which showed the interpretation and inferences of the results by reporting the extracts from the recorded units.

The present study involved a triangulation of researchers, study subjects and data collection techniques, which consisted of a pre-post intervention study combined with a questionnaire, a retrospective study with databases, focus groups with study subjects and researchers with field notebooks, as well as monthly audits with the managers [35].

## Results

### Longitudinal study

In the four hospitals included in the study, 28 falls were recorded longitudinally. The age of the patients was 71.1 ± 14.4 years, 57.1% being male. 39.3% of the falls were from beds with handrails and 50% were visualized by healthcare professionals. The main causes of falls included loss of balance (25%) and loss of strength (21.4%), with the patient's room being the main location (75%). During 2018 (53.6%), and in particular May 2018 (32.1%), the highest number of events was recorded.

Regarding the nurses who attended the falls, the average age was 47.43 ± 8.57 years, the majority being women (82.1%) who worked a morning shift from Monday to Friday (42.9%). 44.4% of the nurses who recorded events reported poor sleep quality and 7.7% reported mild drowsiness. 7.4% of nurses were at risk of emotional pathology and 14.8% had a score corresponding to a moderate evening chronotype. Behavioural and performance data of health professionals (previously mentioned) are pertinent to justify the implementation of interventions. In this respect, one of the interventions proposed to the centres was “monitoring and improvement in the distribution of work shifts among health professionals" (Supplementary material 1).

### Retrospective study

A total of 194 falls were recorded in the institutional registers during the 3 years analysed. The mean age of those who suffered an in-hospital fall was 66.9 ± 17.7 years, 61.9% being male. Regarding marital status, 59.8% were married, 12.4% were single and 2.6% were widowed or separated. 61.9% of the falls were recorded in the referral hospital. In 70.6% (*n* = 137) of all falls, the time of the fall was recorded. Wednesday and Thursday were the days with the highest incidence of falls, with 18.6% each, and Sunday was the day with the lowest number of falls, with 9.3% of the total. Specifically, 32% of the falls occurred during the weekend (Friday-Sunday). The month with the highest number of falls was June (12.9% of falls), and the month with the fewest was December (3.1%). As for the work shift, most falls occurred during the night (22 h to 8 h), with 43.8% (*n* = 85). The shift with the lowest number of falls was the afternoon (15 h-22 h), with 20.6% (*n* = 40). As for the visualization of falls, 46.4% (*n* = 90) were not observed by another person, i.e., the patient was alone, in the absence of a healthcare professional, family member or roommate. The place where most of the falls occurred was in the bedroom (*n* = 111), followed by the bathroom (*n* = 44), and therefore, 80% of the falls occurred in the bedroom environment. The most frequent cause of fall was accidental (35.6%) and 17.5% were due to factors related to the environment. The most frequent position in which the patient was before falling was upright (54.6%). Only 65% of the falls led to injury in the patient, and of these, 40.2% suffered a slight injury and 17.5% an injury that caused incapacity, with incapacity being understood as causing a decrease in their abilities or quality of life, in some cases requiring surgery, reoperation or an extension of their hospital stay. Finally, 35.6% of falls had a critical impact on the patient.

**Fig. 1:**
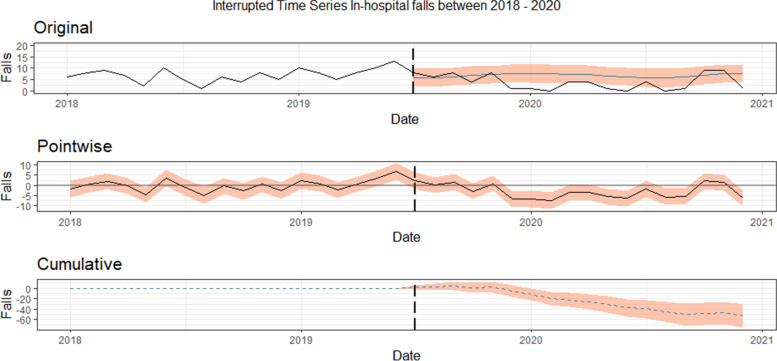
Original and predicted number of falls between January 2018 and December 2020 in all hospitals. Nurse-led intervention was introduced in June-July 2019

Regarding the different study periods, 125 falls occurred pre-intervention (64.4% of the total), and the frequency was significantly lower in the post-intervention period (*n* = 69; 35.6%; *p* < 0.001). During the latter period, the response variable had an average approximate value of 3.83. In contrast, without an intervention, an average response variable of 6.79 would have been expected (95% interval of this counterfactual prediction: 5.54, 8.01). Subtracting this prediction from the observed response yields an estimate of the causal effect the intervention had on the response variable, which in this case was -2.96 (95% interval: -4.17, -1.70), which means that the negative effect observed during the intervention period was statistically significant (Fig. 1). Figure 1 shows three graphs showing the decrease in in-hospital falls after the intervention. Although the downward trend in the “cumulative” graph started in autumn 2019, it should be considered that the first positive result for SARS-CoV-2 in the study setting occurred on 26 February 2020. In fact, during the pandemic there was a reduction in the number of hospital admissions and those admitted may have had more complex therapeutic care and more limited mobility.

Regarding the time of fall, there was an increase in recording after the intervention (64.8% vs. 81.2%; *p* = 0.01). In addition, the recording of non-visualized falls also increased, although not significantly (41.6% vs. 55.1%; *p* = 0.08). It should be noted that differences were found in the incidence of falls occurring during the summer period [June (18.4% vs. 2.9%; *p* = 0.002), July (4.00% vs. 18.8%; *p* = 0.0006), and August (0.8% vs. 8.7%; *p* = 0.004)]. Falls recorded when the patient was in bed with the bedrails up increased in the post-intervention period from 7.2% to 17.4% (*p* = 0.02). Overall, there was a more complete record after the intervention (78.57%; *p* < 0.001).

**Fig. 2:**
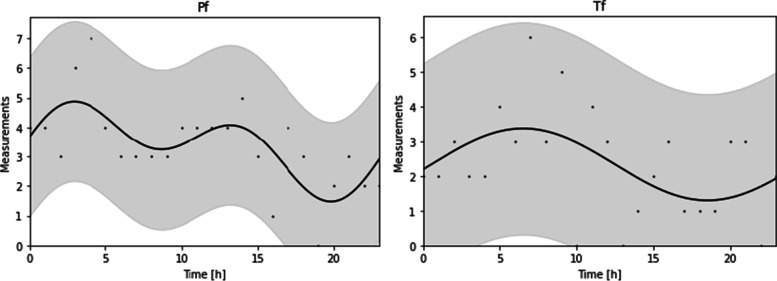
24-h distribution of in-hospital falls pre- and post-intervention

With regard to the time series analysis, in 70.6% (*n* = 137) of the falls, relevant data were obtained to be able to perform this analysis. Table 1 shows the characteristics of the 24-h time patterns according to the characteristics of the falls. With the multi-component Cosinor analysis (Table 1), and after classifying by different variables (intervention, witnessed, localization, cause, position and injury), significant temporal patterns have been obtained, with specific incidence peaks. Specifically, regarding to intervention, Fig. 2 shows that in the pre-intervention period two incidence peaks are observed at 2:55 h and 13:12 h, while in the post-intervention period the main peak is at 6:30 h.

**Table 1:** 24-h patterns of falls according to characteristics (Multi-Component Cosinor analysis)

**Characteristics/categories**	***p*-value**	**Orthophase†**	**Amplitude††**	Bathyphase*	MESOR**
**Intervention**					
**Pre**	0.01	2:55	1.69	19:50	3.17
**Post**	0.05	6:33	1.03	18:32	2.33
**Witnessed**					
**Family or another patient**	0.02	4:13	0.88	16:14	1.87
**Self-documented**	0.05	7:18	1.35	19:18	2.95
**Health professional**	NS				
**Localization**					
**Patient’s room**	0.001	4:31	2.46	21:20	4.24
**Patient’s bathroom**	< 0.001	9:33	1.79	17:06	1.29
**Other localization**	NS				
**Cause**					
**Accidental**	0.04	5:25	1.19	17:23	2.37
**Clinical**	0.05	7:52	0.74	19:53	1.75
**Environment**	0.04	10:45	0.66	22:46	0.91
**Other**	0.04	23:49	0.61	11:49	0.62
**Position**					
**While standing**	< 0.001	8:56	1.62	20:56	2.95
**While sitting**	0.01	14:45	0.54	2:44	0.83
**From bed (rails up)**	< 0.001	3:50	1.47	11:05	1.11
**From bed (rails down)**	0.03	2:21	0.76	14:21	1.12
**Injury**					
**Inconsequential**	0.04	5:05	0.86	17:06	2.25
**Slight**	< 0.001	7:58	1.17	19:59	2.24
**Disabling**	NS				

Explanations: † main peak in the temporal pattern; †† the numerical difference between the peak and valley values; * main valley value in the pattern; **the estimated rhythmic mean of the time series

### Qualitative study

A total of 26 members participated in five focus groups (four groups consisting of 3 nurses and two assistant nurses; and one group with three nurses and three assistant nurses). All except two of the participants were women. The thematic categories obtained were related to: i) concept of a fall: personalized care and prevention; ii) difficulty of care: accompaniment and COVID-19 pandemic; and iii) causes of lack of registration: proposals for improvement and training received.

#### Concept of a fall: personalized care and prevention

The focus group participants reported that they usually recorded falls, although they expressed difficulties in detecting in-hospital falls because there were disagreements on the concept of what constituted a fall.

[Healthcare professional – P1: I don't think we don't have any falls, but if we observe them, they are registered, and we fill in an adverse event document].

[P1: Sometimes it could be considered a fall, but with some incidents, it’s hard to decide, so in the end they are not recorded].

[P2: Of course, maybe she slips off the couch and falls on the floor, and those cases haven't really been considered].

The participants reported certain factors that in their view increase the risk of patient falls in the unit, including disorientation, over-medication for sedation, mobilization to and from the toilet, and the lack of continuity of the nursing professionals in the unit.

[P3: And it's a shame, too, because you stuff them with medication. The next day, the older person is exhausted—he is an older person and he’s totally worn out. This is detrimental to the grandfather's activity, to his reactivity, and he’s active again at night] [P4: You wanted him to go to sleep and now you want him to wake up and then you start putting him on medication to get him to wake up]. [P5: It’s true that the continuity that you can have with a patient by getting to know him avoids many problems. If you don't know them, there are things that… you can be careless and that’s obvious when you’re one day in one place and another day in another, well…; maybe a person who has more work continuity can avoid that fall because they know them, and you (faced with that situation at work) get distracted, and they can fall]. [P3: When the oxygen drops in the blood, they also get a little dizzy and so this often makes them… they think they are fine, they go to the bathroom, maybe they take off the oxygen and they get dizzy… It’s also true about the floor, the screens… you have to insist on that with them a lot, and keep an eye on them, and tell them "don't take off your oxygen to go to the bathroom", "don't go alone", "let us know when you’re going", because many of the falls in the bathroom are due to a lack of oxygen].

Preventive measures could be aimed at structural and staffing improvements. [*P2: And maybe we could also have more health workers who can help us with mobilizations and all that, that too] [P2: Well, that's a difficult thing to do, we bought the patient transfer discs, to see if that would make it easier, but of course that's one of the dangerous manoeuvres, because either you’re very sure that you’re going to be able to handle the patient, or…].*

#### Difficulty of care: accompaniment and COVID-19 pandemic

The focus groups reported the fact that patients were unaccompanied by family members during the Covid-19 pandemic, although according to the participants, this made no difference. *[P3: It's the same. A patient, as the beds have handrails, those who are more… with more predisposition, maybe to get up, or more dependent, they also have their own handrail and this makes them get up less]. [P2: But Covid hasn’t made much difference… I don't think so, anyway, for any of us’].*

In fact, they claim that unaccompanied patients are no more at risk of falling than those accompanied by family members. *[P7: The thing is when the patient is alone, that's when he falls the least, because we don't even get to pick him up. He gets up on the couch on his alone, you know? And he doesn't dare to do it alone either]. [P6: So many times they say, come on, I'll help you and maybe later they can't, because the wife… and… he falls…].*

#### Causes of lack of registration: proposals for improvement and training received

In addition to issues referring to the concept of a fall and the presence or absence of accompaniment during the occurrence of the fall [*P3: If they are not accompanied by a carer they don't usually, they don't usually get up, so the carer always warns you. So, yes, they do slip. But they don't fall, they don't fall*], other factors such as difficulties in accessing the registration application, fear of registration, lack of time and staff have also been reported (Table 2).

**Table 2:** Difficulties in recording falls, according to participant’s perceptions

Units of meaning	Topic
*“I heard, come on, I haven't registered any because I haven't seen anyone fall, but I've heard that it's really difficult and that you have to do I don't know what… and then you have to… that's really difficult” Maybe the most difficult thing is finding the place to enter. Entering the page…”*	Non-friendly information system
*I think that knowing that it's a confidential thing, that your name doesn't come out. Because maybe there are people who don't register it for fear of what will happen to them later because the patient has fallen.”*	Fear of registration
*But of course, you think if there is a fall, someone’s going to show you how to register the fall, but nobody trains you. It was a bit on the fly, so if during the rest of your colleagues' shifts there was no fall, nobody teaches you]*	Lack of time and personnel
*So, you train people and then you get very new people who don't have the training… so it's starting all over again, and it's a bit exhausting*	Lack of awareness and motivation

As regards preventive measures, the professionals suggest that there should be easier, direct access to the computerized registration application, online training on the use of the application, confidentiality in the registration of falls and that new staff should be provided with clear instructions on how to register care incidents and adverse events (Table 3).

**Table 3:** Preventive measures to encourage the recording of falls as perceived by professionals

Units of meaning	Topic
*“Yes, direct access would be really nice, so you don't have to… go to the website because there are people who don't know where the hospital website is”*	Direct access to the electronic application for registration
*“It’s really complicated, because it’s very difficult to attend in person. So, it’s online training, and each person has to fit it in to their busy schedule.”*	Online training on falls recording
*“Saying that it’s confidential and knowing where it’s registered. I think that would improve things a bit more”*	Confidentiality in the registration of falls
*“So, that's why I tell you that, from the beginning, for all new people, it would be great if, just as you get a uniform and an accreditation with an access code, you get clear instructions one morning about the care module, assessment, care plan, discharge care…”*	Initial training of junior professionals

## Discussion

The present study, following a mixed-model approach, shows the findings of a multicentre nurse-led intervention in the prevention of in-hospital falls. The mixed-model approach was implemented consecutively, as inconsistencies in the information obtained from the study subjects in the pre-post intervention approach became apparent, which led to the retrospective study. In turn, as the inconsistencies continued, focus groups were set up. This implementation process confirmed the rigorous methodology followed in the project. In fact, the final findings were no less discouraging, noting, for example, the absence of records in some cases and their completeness in others, or the different ways the professionals understood the concept of a fall (which, in turn, resulted in the presence or absence of records, as well as affecting their quality).

Inconsistencies were mainly observed during the monthly audits with the managers of the centres. In the pre-intervention phase, the number of falls recorded in the study's own register was lower than the number of falls recorded on the institutional adverse event reporting database, Consequently, the research group considered the implementation of a mixed-methods model, in which the qualitative approach could provide an answer to the inconsistencies.

With regard to the nursing-led measures, the chronobiological perspective was taken into account, i.e. the time the fall occurred (clock time and calendar date). Previous studies by the research group have highlighted the importance of knowing the time of day in order to design more comprehensive preventive measures [20–22, 27].

Chronopreventive measures were implemented in four hospitals, each with a wide population coverage. Taking into account the multi-causality of the falls and the information obtained from the registers and monthly audits with managers, organizational, educational and behavioural chronopreventive measures were agreed upon with each hospital. Each centre was committed to implementing the measures it considered appropriate, as they implied changes at organisational or operational level. Although this limited the generalisability of the results and made it difficult to know the effect of each intervention, this approach allowed tailoring the intervention to the centre and its characteristics, which could lead to greater adherence.

However, the use of an interrupted time series analysis, a novel analysis in this field, has provided insight into the possible effect of interventions. Although factors such as the COVID-19 pandemic may have played a role, a 2.96% reduction in the number of falls was observed. In addition, there was an increase in the number of records completed correctly and in information about the time of the fall.

Last but not least, due to the inconsistencies between the data obtained by the different study designs, one of the secondary objectives emerged, which was to address the under-recording of falls, as well as to raise awareness of the importance of carrying out a coherent recording of falls, without fear, idiosyncrasies or passivity, but in a responsible way, as this will enable health policies to be directed towards the patients and their safety. Some studies [9, 20] have reported that health professionals underestimate falls; in fact, although they state that there are high incidences of falls, most of them are not recorded. These data coincide with the perceptions of healthcare professionals in the focus groups. Other actions to improve include both the need to train health professionals on the definition of falls (what is or is not understood by a fall) and improving nurses’ accessibility to adverse event recording systems.

As in the present study, in previous studies carried out by the research group [9, 21], the possible presence of the Hawthorn effect was considered. To mitigate the such effect, participants were randomized selected and healthcare professionals with a management role in the four hospitals were not considered. We intended that participants would not be aware that they were being observed, although researchers are aware that this effect is always present in this type of study.

In addition, this study addresses both patient and healthcare professional safety, as nurses provide 24-h health care, as well as controlling the environment. As reported by other authors [36, 37] and the professionals from the different focus groups, the temporal patterns associated with the patient's environment and how they affect the occurrence of an event such as a fall [38] are no less important than the suitable provision of care, through shift management of shifts, working conditions, patient-nurse ratios and other variables [39], which may impact on patient recording and care. It is therefore clear that the results obtained in this study reflect the need to implement health policies where patient safety is a priority.

There are several limitations to the manuscript. Firstly, falls were recorded by healthcare professionals, so several intrinsic risk factors may have been undocumented and unrecorded, which could confound the accuracy and interpretation of the results. To counter this effect, data triangulation has been used to cross-check information (different data sources, use of multiple observers or methodologies). Secondly, the results of this study may be of limited validity due to the presence of the COVID-19 pandemic which impacted hospital management dramatically. Thirdly, although efforts have been made to make the recording systems more easily accessible and user-friendly, it was found that in some cases this had not happened, which may have influenced the prospective recording of falls. Fourthly, each center decided which interventions to include; this may have limited the generalisability of the intervention and also the potential impact of the intervention.

## Conclusions

The implementation of a program of nurse-led chronopreventive measures in several hospitals seems to have improved patient safety. Organizational, educational and behavioural measures, as well as a time-sensitive falls analysis, have resulted in a more comprehensive recording of in-hospital falls. Furthermore, although the project was conducted during the COVID-19 pandemic, a reduction in the number of falls was observed in all the hospitals analysed.

The present study has served as a basis for rethinking the preventive measures implemented in these hospitals and their clinical practice guidelines, and steps to improve in these areas are already underway.

## Supplementary Information

**Additional file 1: Supplementary Material 1.** Nurse-led chronopreventive measures proposed to the four centres.

## Data Availability

The datasets used and/or analysed during the current study are available from the corresponding author on reasonable request.
