# Supplementary material for: CHRONOFALLS: A multicentre nurse-led intervention in the chronoprevention of in-hospital falls in adults
Source: BMC Nurs. 2023 May 5;22:149. doi: 10.1186/s12912-023-01322-9 (PMC10159679; doi:10.1186/s12912-023-01322-9)
Supplement: Supplementary file 1 — Additional file 1: Supplementary Material 1. Nurse-led chronopreventive measures proposed to the four centres. [file 12912_2023_1322_MOESM1_ESM.docx]

Supplementary Material 1. Nurse-led chronopreventive measures proposed to the four centres.

*1.- Organisational elements:*

- Posters in nursing control areas advising on the need to review medication on admission, and periodically through patient and treatment follow-up. Medication review.
- Monitoring and improvement in the distribution of work shifts among health professionals.
- Identifying and modifying equipment and features in the hospital environment that may contribute to the fall risk (anti-slip tapes, elimination of architectonical barriers,...) both for the patient and for the health professionals.
- Ergonomic structure of hospital beds.
- Patient risk management procedures:
- Alternatives to constraints and/or restrictive elements.
- Assessment of the opportunity and/or need and/or usefulness of restraints.
- Increased number of nursing visits to the patient.
- Promotion of safe mobility and bathing practices based on specific clinical guidelines (Guía Fase para la prevención y actuación ante una caída. Ministry of Health. Junta de Andalucía. 2017)
- Adequate lighting in spaces: patient room, corridors, nursing control, WC....
- Assessment of the opportunity and/or need and/or usefulness of placing handrails on beds.
- In lying position, beds, stretchers and wheelchairs must have the brake on.
- Reporting of falls or potential fall risk in the Incident Reporting System.
- Identifying the locations of falling hazards in the hospital. Approach strategy for their modification (e.g. continuous flooring without paving tiles ....).
- Multidisciplinary assessment of risk factors related to in-hospital falls.
- Discussion in the Patient Safety Committee of the incidents related to in-hospital falls and implementation of the agreed improvement measures.

*2.- Educational elements:*

- Training on the correct mobilization of the bedridden patient in different positions: bed-to-stretcher, bed-to-chair and vice versa; as well as transfer methods.
- Training on alternatives to constraints and/or restrictive elements

*3.- Behavioural elements for patients:*

- Patient support and instruction in preventive health measures:
- Wearing appropriate footwear and, where necessary, including orthoses.
- Training in the use of walking aids.
- Repeated reminders to patients of the need to notify the team for any situation (getting up/lying in bed, going to the toilet/shower, walking in the corridors, ...).

*4.- Behavioural elements for healthcare professionals:*

- Taking special precautions to prevent falls by healthcare professionals at mealtimes and during the different transfers that can be carried out on the patient:
- Identifying proper circumstances for the use of handrails.
- Identifying and modifying equipment and other factors in the hospital environment that may contribute to the risk of falls (anti-slip tapes, elimination of architectural barriers...) for both patients and healthcare professionals.
- Patient risk management procedures:
- Post-fall monitoring.
- Alternatives to constraints and/or restrictive elements.
- Increased number of nursing visits to the patient (especially at peak fall occurrence times).
- Promotion of safe mobility and bathing practices based on specific clinical guidelines (Guía Fase para la prevención y actuación ante una caída. Ministry of Health. Junta de Andalucía. 2017)
- Bed height control in at-risk patients.
- Performing medication checks and reviews on patient admission.
- Reporting of falls or potential fall risk in the Incident Reporting System.
